# A human lower-limb biomechanics and wearable sensors dataset during cyclic and non-cyclic activities

**DOI:** 10.1038/s41597-023-02840-6

**Published:** 2023-12-21

**Authors:** Keaton Scherpereel, Dean Molinaro, Omer Inan, Max Shepherd, Aaron Young

**Affiliations:** 1 Woodruff School of Mechanical Engineering, Georgia Institute of Technology, Atlanta, GA 30332 USA; 2 Institute of Robotics and Intelligent Machines, Georgia Institute of Technology, Atlanta, GA USA; 3 School of Electrical and Computer Engineering, Georgia Institute of Technology, Atlanta, GA 30332 USA; 4 Department of Mechanical & Industrial Engineering, Northeastern University, Boston, MA 02115 USA; 5 Bouve Department of Physical Therapy Movement and Rehabilitation Sciences, Northeastern University, Boston, MA 02115 USA

**Keywords:** Mechanical engineering, Biomedical engineering

## Abstract

Tasks of daily living are often sporadic, highly variable, and asymmetric. Analyzing these real-world non-cyclic activities is integral for expanding the applicability of exoskeletons, protheses, wearable sensing, and activity classification to real life, and could provide new insights into human biomechanics. Yet, currently available biomechanics datasets focus on either highly consistent, continuous, and symmetric activities, such as walking and running, or only a single specific non-cyclic task. To capture a more holistic picture of lower limb movements in everyday life, we collected data from 12 participants performing 20 non-cyclic activities (e.g. sit-to-stand, jumping, squatting, lunging, cutting) as well as 11 cyclic activities (e.g. walking, running) while kinematics (motion capture and IMUs), kinetics (force plates), and electromyography (EMG) were collected. This dataset provides normative biomechanics for a highly diverse range of activities and common tasks from a consistent set of participants and sensors.

## Background & Summary

Human movement in daily living varies from consistent and steady activities, such as walking and running, to more dynamic movements that vary by person and situation, such as lifting, lunging, or side-stepping. Not only do the biomechanics of these motions vary, but the actual activities are sporadic. Humans stop, start, and switch tasks rapidly, often with transitions that are difficult or impossible to define. This variety is reflective of the diverse and dynamic goals for human movement, the flexibility of the human musculoskeletal system^1^, and the many environmental factors that impact movement^2^. The diversity in human activity is evidenced by the lack of continuous movement in everyday activities. The most common walking bout lasts only about 4 steps, and 75% of both walking and resting bouts last less than 70 seconds^3^. Orendurff *et al*. demonstrated that gaits of daily living consistently reflect a dynamic approach to movement; one where more emphasis is placed on maneuverability than on highly efficient consistent walking^3^. Despite this fact, the vast majority of biomechanics research has focused on long-duration, steady-state walking bouts.

Open-source datasets to date have largely centered around the most common cyclic and steady-state activities, which can be easily segmented and averaged across strides. Winter’s seminal dataset characterizing human biomechanics focused only on the time-repeatable task of level-ground walking^4^. Since then, publicly available datasets have begun to increase the variety of data available by including more walking speeds^5–8^ and more participants^9,10^. These datasets have not been limited to level-ground walking but have included other time-repeatable tasks including running^11,12^, walking on stairs and ramps^13–18^, sitting and standing^13,14,19,20^, heel and toe walking^21,22^, and even walking on irregular terrain^23,24^. In addition to increasing the task space, other sensor modalities have been explored, such as muscle specific information measured through electromyography (EMG)^9,10,13–18,21,22,25,26^. Research from Camargo *et al*. has filled a critical gap by providing a single dataset of the same participants performing multiple terrain conditions at multiple speeds with consistent and comprehensive sensors^15–18^. Reznik *et al*. also filled some of these gaps with their public dataset, including additional ambulation mode transitions, sit-to-stand movements, and constant acceleration tasks^19,20^. Still, there exists very limited publicly available data for transitions^13–20^, and non-cyclic dynamic tasks that represent the variability of daily life are largely absent. The state of current open-source datasets in literature is summarized in Table 1 for cyclic activities and Table 2 for non-cyclic activities.

**Table 1:** Cyclic Tasks in Open-source Biomechanics Datasets for Able Bodied Individuals.

Metadata			Common Cyclic Tasks						Uncommon Cyclic Tasks			
	# Participants	Age Range	Walk (m/s)	Run (m/s)	Stair Ascent (mm)	Stair Descent (mm)	Incline (°)	Decline (°)	Miscellaneous (Heel/Toe Walk)	Walk Backwards	Weighted Walk	Calisthenics
Winter 1983^4^	1		SS (1 speed)									
Moore 2015^7,8^	15	19–32	~0.8–1.6									
Fukuchi 2017^11,12^	28	19–51		2.5–4.5								
Fukuchi 2018^5,6^	42	21–84	SS (8 speeds)									
Hu 2018^13,14^	10	23–29	SS (1 speed)		197	197	10	10				
Lencioni 2019^21,22^	50	6–72	SS (5 speeds)		180	180			x			
Schreiber 2019^9,10^	50	19–67	SS (5 speeds)									
Moreira 2021^25^	16	20–27	0.28–1.1									
Camargo 2021^15–18^	22	19–33	0.5–1.85		102–178	102–178	5.2–18	5.2–18				
Reznick 2021^19^	10	20–60	0.8–1.2	1.8–2.4	97–162	97–162	5, 10	5, 10				
**Our Dataset**	**12**	**18**–**30**	**0.4**–**1.8**	**2.0**–**2.5**	**152**	**152**	**5, 10**	**5, 10**	**x**	**x**	**x**	**x**

*SS indicates self-selected walking speed.

**Table 2:** Non-Cyclic Tasks in Open-source Biomechanics Datasets for Able Bodied Individuals.

	Step over	Sit-to-stand	Turn	Squats	Lift weight	Jump across	Poses	Cutting	Lunges	Ball Toss	Step Ups	Curb	Jump in place	Others
Winter 1983^4^														
Moore 2015^7,8^	x													
Fukuchi 2017^11,12^														
Fukuchi 2018^5,6^														
Hu 2018^13,14^		x												
Lencioni 2019^21,22^														
Schreiber 2019^9,10^														
Moreira 2021^25^														
Camargo 2021^15–18^			x											
Reznick 2021^19,20^		x	x											
**Our Dataset**	**x**	**x**	**x**	**x**	**x**	**x**	**x**	**x**	**x**	**x**	**x**	**x**	**x**	**x**

Dynamic, non-cyclic activities in biomechanics literature have received attention due to their relevance to rehabilitation, sports, and injury prevention as well as their prevalence in everyday life. Activities such as sit-to-stand and obstacle avoidance are important for independent daily living and the ability to maintain stability^27,28^. Other studies have examined the biomechanics of highly dynamic activities, such as cutting^29,30^, jumping^31^, lifting^32,33^, lunging^34^, and squatting^35^ and their relevance for athletes in preventing injury and promoting rehabilitation. These motions differ significantly from walking but directly and indirectly have bearing on the majority of movements that an individual might encounter in daily life. Despite the relevance to daily living, there are no open-source datasets containing many of these tasks, let alone collecting all these types of maneuvers with continuous time series data and corresponding wearable sensors.

This paper presents an open-source biomechanics dataset that includes a wide range of both dynamic non-cyclic tasks and cyclic tasks. It represents the most diverse open-source set of biomechanics data across weight bearing lower limb tasks available in the literature. This includes the specific non-cyclic tasks mentioned above as well as unique tasks that involve perturbations from external objects. Additionally, representative tasks are included from cyclic walking, such as level ground walking, stairs, and inclines as well as unique cyclic tasks such as heel and toe walking and backward walking. For each of these tasks, kinematics from motion capture as well as real and simulated IMUs are supplied. Kinetics from force plates and the resulting joint moments calculated through OpenSim^36^ are also included, as well as virtual insole data. Finally, time-synchronized EMG from eight muscles on each leg is provided. The data from this complete suite of wearable sensors are a valuable contribution to the literature for many applications, such as machine learning, wearable device design, controller development, health monitoring, and human modeling.

## Methods

In this study, 3D biomechanics from marker-based motion capture and force plates, as well as wearable signals from IMUs and EMGs, were collected for a diverse range of ambulatory tasks as well as numerous non-cyclic tasks. *Virtual sensors* were created to increase the usefulness of the dataset by providing data for sensors that were not included in this study, such as pressure insoles and foot IMUs. This also provides data for comparison, replication, and advancement of research with simulated sensors^37,38^.

### Participants

Twelve able-bodied participants (7 males, 5 females, mean ± SD: age = 21.8 ± 3.2 years, height = 176.7 ± 8.6 cm, weight = 76.9 ± 14.4 kg) were recruited to participate in a single-day gait analysis study. For inclusion, the participants had no history of neurological injury, gait pathology, or cardiovascular condition that would limit their ability to ambulate for multiple hours, up and down steep inclines and stairs, and participate in fatiguing exercises. Each participant gave informed written consent to participate in a Georgia Tech Institutional Review Board-approved study under IRB H17240.

### Performed tasks

**Fig. 1:**
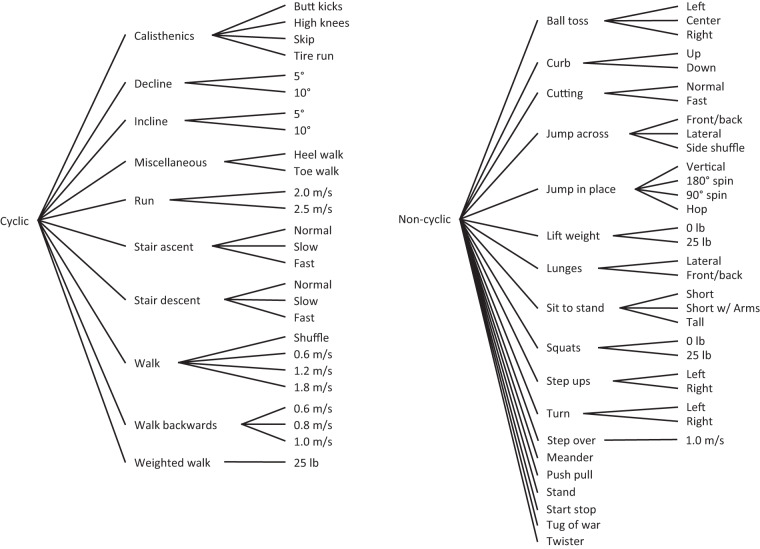
A representation of the cyclic and non-cyclic tasks broken into their constituent conditions.

Tasks were selected to provide large inter-joint variability in kinematics and kinetics, and to provide examples of similar kinematics but differing kinetics. The tasks can be split into several different subcategories within the broad categories of *cyclic* and *non-cyclic* as shown in Fig. 1. A full list and description of how each of the activities was performed is given in Supplementary Table 1 and in the videos (not taken during actual collections) included within the dataset.

### Motion capture

**Fig. 2:**
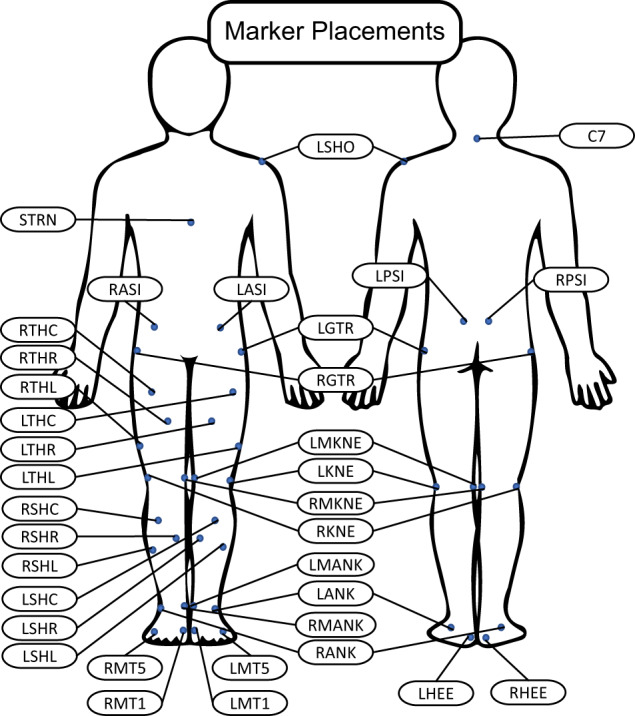
The modified Helen Hayes marker set used for data collection.

Retroreflective markers were placed based on a modified Helen Hayes marker set^15^ including markers on the medial malleoli, medial femoral condyles, greater trochanters, and posterior superior iliac spine (Fig. 2 abbreviations detailed in the dataset).

Motion capture data were collected at 200 Hz (Vicon. Ltd., Oxford, UK) using 29 cameras overhead and 4 ground cameras. Gap’s were filled (with rigid body fill and pattern fill) using an automatic pipeline (max gap length 200 frames) to fill some gaps and then manual examination was used to verify and fill the remainder. Trajectories were then low pass filtered (zero-lag 6 Hz 5th order Butterworth filter^15,19,39^) before processing in OpenSim. Subject-specific models were based on the OpenSim gait2354 model^40^. They were scaled based on anatomical landmarks and participant mass as measured via force plate data during static trials. During scaling, mass distribution between segments was preserved and main anatomical landmarks were used for pose positioning (highest weight: LASI, RASI, LPSI, RPSI, RKNE, LKNE, RMKNE, LMKNE, LANK, RANK, LMANK, RMANK, C7; lower weight: LGTR, RGTR, LMT1, LMT5, LCAL, RCAL, RMT1, RMT5). Sagittal joint angles were required to be within ±5° of 0 for neutral standing static trials to verify that the model matched the human’s pose.

### Ground reaction forces

Ground reaction forces were collected with both an instrumented treadmill (Bertec Corporation, Columbus, Ohio) as well as over ground force plates (Bertec Corporation, Columbus, Ohio) depending on the task. Force plate data were collected at 1000 Hz and then low pass filtered (20 Hz 5th order Butterworth zero-lag^41,42^) and clamped to zero for forces less than 20 N. Force plates and the treadmill were re-zeroed upon configuration changes. Forces were split into steps based on the 20 N threshold (except for the treadmill trials that included running in which a 50 N threshold was applied due to higher noise) and then were automatically assigned to their respective feet based on matching marker and center of pressure data in the global coordinate system.

### Joint angles and velocities

The subject-specific models were used to calculate inverse kinematics based on marker data where each marker was weighted equally. In the event of a marker falling off, a new static calibration was performed and then the marker placement on the model was shifted to match the new placement without changing the scaling of the skeletal model (details on specific trials are provided in the dataset). Inverse kinematic calculations within OpenSim were performed to estimate joint angles based on marker positions. The unfiltered kinematics are provided in the dataset. However, for any further steps such as inverse dynamics or joint velocities, a filter (6 Hz lowpass forward-reverse Butterworth filter) was applied^36^. The hip, knee, and ankle velocities were computed from the filtered inverse kinematics using central finite differencing (MATLAB’s gradient function).

### Joint moments and powers

Filtered inverse kinematic data were used in conjunction with ground reaction force data to calculate inverse dynamics. Steps not on the force plates were marked as NaN for inverse dynamics from heel strike of the first step off the force plates to toe off of the last step before returning to the force plates. Joint moments were then lowpass filtered using a forward-reverse 5th order Butterworth filter with a cutoff frequency of 6 Hz. These moments were then multiplied by the above velocities to compute joint powers.

### Electromyography

EMG data were collected at 1259 Hz bilaterally (Trigno Avanti Wireless EMG, Delsys, Natick, MA) and upsampled to 2000 Hz (default for Delsys digital sensor data acquisition in Vicon Nexus). EMG data was synched with the motion capture data using the Delsys trigger module (Delsys, Natick, MA). EMG were recorded from the following muscles bilaterally: gluteus medius (GMED), gluteus maximus (GMAX), gracilis (GRAC), biceps femoris (BF), vastus lateralis (VL), rectus femoris (RF), tibialis anterior (TA), medial gastrocnemius (MGAS). Seniam guidelines were used to determine EMG placement^43^. The location was prepared with alcohol and then the sensor was adhered to the skin using double sided adhesive. EMG data are provided as raw unfiltered data.

### IMUs

**Fig. 3:**
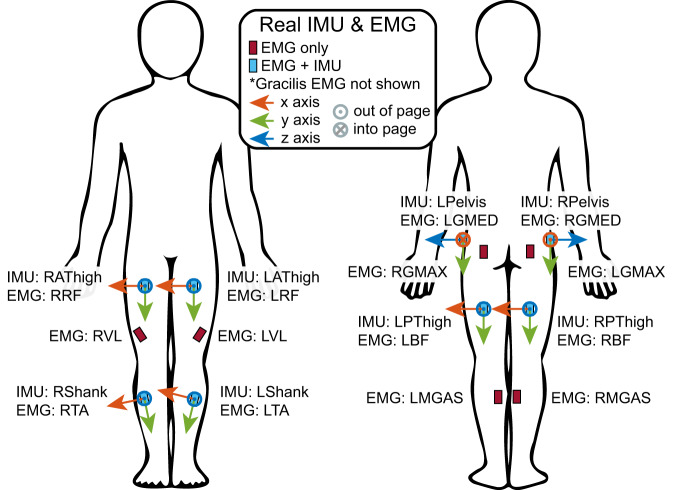
The orientations, placement, and axes definitions for physical inertial measurement units (IMUs) and surface electromyography sensors (EMGs).

IMU data (Trigno Avanti Wireless EMG, Delsys, Natick, MA) from experimental sensors were collected at 148.6 Hz at several locations in combination with EMG. Due to these data coming from the same sensor package as the EMG, the system automatically upsampled to 2000Hz to match the EMG data. In the dataset, IMUs have been downsampled back to 200 Hz to better reflect the collection frequency and to match the rest of the mechanical data. IMUs were collected bilaterally at the same locations as EMGs on the gluteus medius, biceps femoris, rectus femoris, and tibialis anterior. The placement for EMGs and actual IMUs is presented in Fig. 3.

### Virtual inertial measurement units (IMUs)

**Fig. 4:**
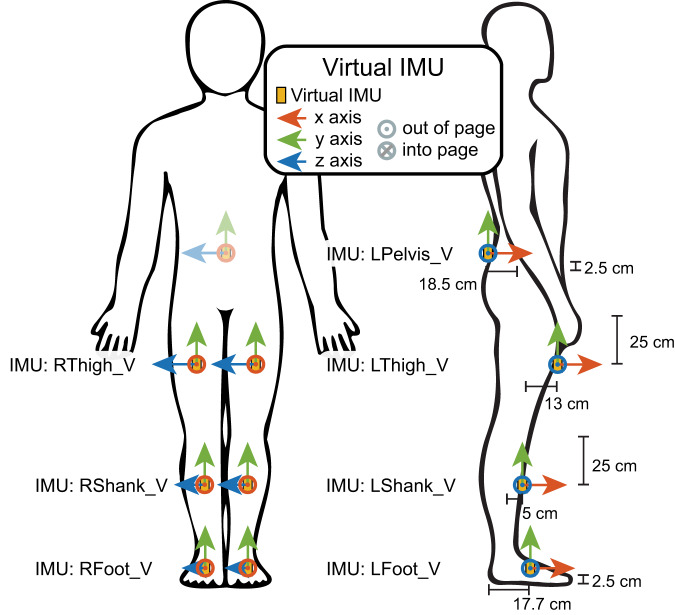
The orientations, placement, and axes definitions for the simulated inertial measurement units (IMUs).

Filtered kinematics in OpenSim were used to calculate transformation matrices to transform segment positions into global frame coordinates for each individual time step. Simulated IMUs were positioned on the pelvis, thigh, shank, and foot bilaterally as shown in Fig. 4 with all of the medial-lateral positions located at the center of their respective segments.

These above positions in the local frame were converted via the transforms to the position in the global frame at each time point. Then these positions were differentiated twice to find acceleration. Gravity was then included along the proper axis. The rotational transformation matrix was again used to find the rate of rotation at each point to calculate gyroscope measures. This process is outlined and verified in Molinaro *et al*.^38^.

### Virtual insoles

These same transformation matrices were inverted and used to convert the global force plate measures into the foot reference frame. This provides forces with respect to the foot for both vertical ground reaction force as well as anterior-posterior shear and mediolateral shear. The center of pressure was calculated in a similar fashion based on the position of force application on the force plate converted to global coordinates and then transformed to the foot reference frame based on the instantaneous transformation matrix. This provides an anterior-posterior center of pressure as measured from the back of the foot and a medio-lateral center of pressure with respect to the center of the foot.

### Summary data visualizations

#### Task specific joint dynamics

**Fig. 5:**
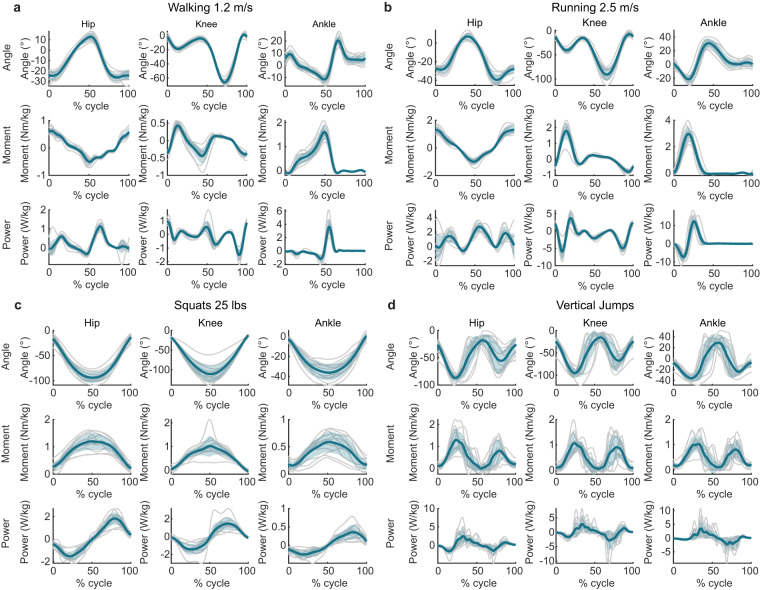
Representative biomechanics for a selection of tasks averaged across participants. Walking at 1.2 m/s (**a**) and running at 2.5 m/s (**b**) were segmented by gait cycle while squats with a 25 lb weight (**c**) and jumps (**d**) were segmented by a pelvis velocity threshold. Figures for all of the tasks and segmentation information is contained in the dataset.

A summary of the biomechanics for several tasks that can be segmented is presented below in Fig. 5 while the complete set of figures is included directly within the dataset. Each figure consists of nine subplots with each column representing the three joints (hip, knee, and ankle) in the sagittal plane and each row representing angle, moment, and power respectively. Individual participant lines are included in grey while the across participant average and standard deviation are presented in color. In these figures, hip extension is positive, knee extension is positive, and ankle plantarflexion is positive.

#### Motion space comparison of cyclic and non-cyclic tasks

**Fig. 6:**
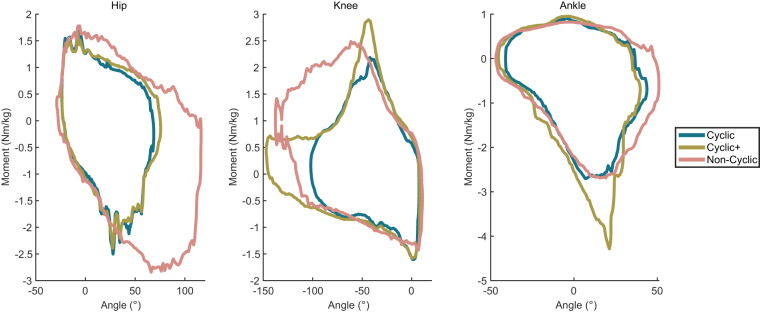
Moment angle representation of the conventional cyclic (level ground, ramps, and stairs), augmented cyclic (all collected cyclic tasks including the common cyclic tasks as well as unique cyclic tasks such as backward walking and high knee walking), and non-cyclic (such as sit-to-stand and cutting) domains.

To visually demonstrate the differences between cyclic and non-cyclic domains and further illustrate the uniqueness of these non-cyclic activities, the data were examined within the moment and angle regime, thus quantifying the kinematic and kinetic scope of data across all tasks including those that could not be segmented. Joint moment and angle data over time were combined for three distinctions of activities: 1. *Cyclic*: the most commonly studied activities consisting of walking on stairs, ramps, and level ground 2. *Cyclic*+: all collected cyclic tasks (including running, backward walking, heel & toe walking, etc.). 3. *Non-cyclic*: the 20 non-cyclic tasks described above. A kernel density estimator^44^ was then passed over the data and the largest continuous contour containing 99.5% of the data across all participants was created to represent the motion space in the kinematic (as represented by angle) and kinetic (as represented by moment) domains. These plots are presented in Fig. 6 for the hip, knee, and ankle.

This demonstrates that these non-cyclic activities cover a new domain within both the kinematic and kinetic regime of human movement specifically for the hip and the knee.

#### Task specific moment-velocity requirements

**Fig. 7:**
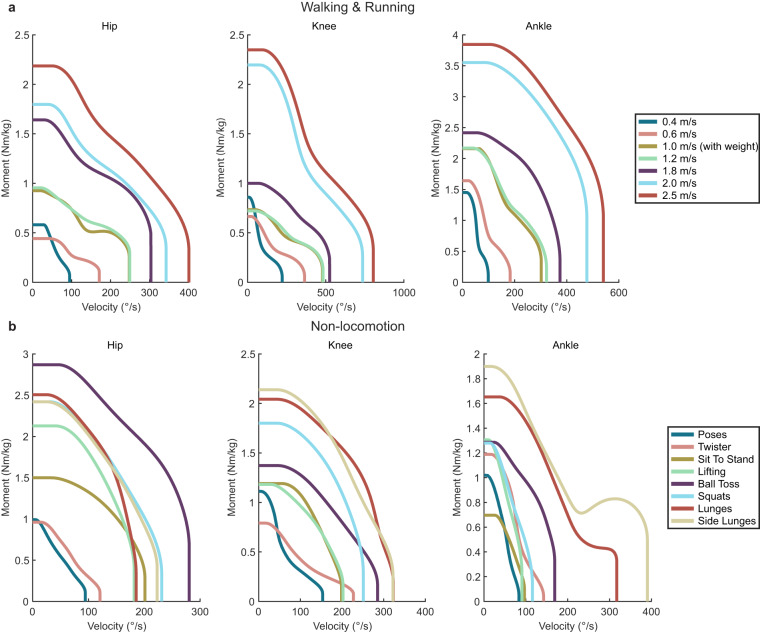
The maximum biological moment and velocity demand for walking and running (**a**) as well as for a series of the non-cyclic, non-locomotion tasks (**b**). Figures for all of the tasks are contained in the dataset.

As a separate visualization to examine specific tasks, a similar approach was taken to determine the biological moment-velocity demands for specific tasks. This provides the biological analog for a torque-speed curve for electric motors. As above, a kernel density estimator was used to form a continuous non-parametric estimate for density and then a continuous contour for 95% of the data was taken to capture the maximum biological moment and velocity demand for each task. This particular visualization highlights the required operating ranges and specifications for designing wearable devices to handle extremes of human tasks. An example of several of these plots are included in Fig. 7 and the rest are included within the dataset itself.

This visualization highlights how the power requirements of different tasks vary, thus further emphasizing the differences between the cyclic and non-cyclic domains.

## Data Records

The data is given in four separate folders: Processed Data, Raw Data, C3Ds, and Segmentation. The data can be found at the SMARTech repository^45^.

### Processed data

**Fig. 8:**
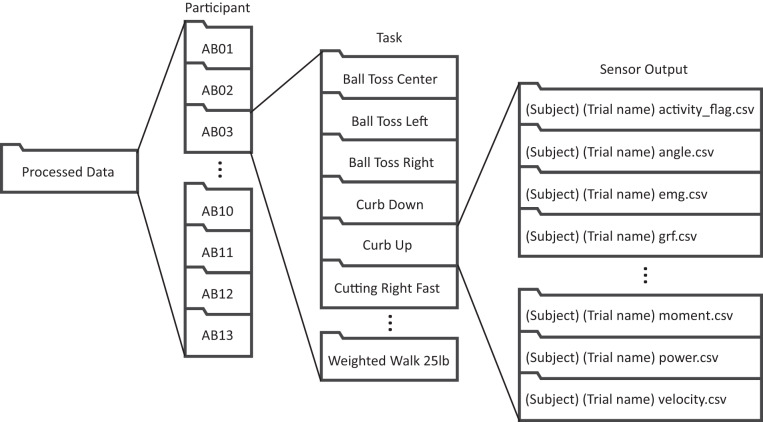
A visual representation of the processed data folder structure.

Processed data are listed by participant first then by trial type. Each folder contains.csv files for the measurements listed above. This is summarized in Fig. 8.

The breakdown of the csv files listed for each task of each participant are listed in Table 3.

**Table 3:** Output File Types for Each Task.

File	Sampling rate (Hz)	Units	# of columns	Contents description (the first column of every file is a time column in seconds)
_activity_flag.csv	200	boolean	3	The first two data columns are columns of zeros (off) and ones (on) for which the participant is performing the given activity (on) for left and right respectively.
_angle.csv	200	degrees	13	The first 6 data columns are for the right leg and consist of 3 angles for the hip (flexion/extension, adduction/abduction, rotation), 1 for the knee (flexion/extension), 2 for the ankle (dorsiflexion/plantarflexion, eversion/inversion). The final 6 columns are broken down in the same way as above for the left leg.
_emg.csv	2000	volts	17	The data columns come in pairs of columns for left and right EMG raw values (noted L and R). These are for each of the muscle locations listed in the methods section: tibialis anterior (TA), rectus femoris (RF) biceps femoris (BF), gluteus medius (GMED), medial gastrocnemius (MGAS), vastus lateralis (VL), gracilis (GRAC), gluteus maximus (GMAX).
_insole_sim.csv	200	COP (meters), Force (Newtons)	11	The data columns are for two quantities: center of pressure (COP) and force. These are in the reference frame of the foot. The first two data columns are for the right foot COP as measured from the back of the calcaneus for the anterior/posterior direction (AP) and the center of the foot for the medial/lateral direction (ML). The following 2 are for the left COP. The next 3 are for ground reaction forces converted to the foot frame for vertical forces (Vertical), mediolateral (ML), and anterior/posterior (AP) with positive directions as defined by the axes in the methods figure. The last three columns are for forces on the left foot.
_grf.csv	200	COP (meters), Force (Newtons)	13	As above, the data columns are for two quantities: center of pressure (COP) and force. These are in a global coordinate frame and thus the X and Z directions change with the orientation of the person. The first three columns are for right foot force in X, Y (vertical), and Z followed by the COP in X, Y, and Z. This is then repeated for the left leg.
_imu_real.csv	200	Accel (m/s^2^), Gyro (degrees/s)	49	The data columns consist of pairs of six columns from each of the IMUs: acceleration in the X, Y, Z direction and gyro in the X, Y, and Z direction. The sensors begin with pairs for left first and then right for the four locations: shank, anterior thigh (AThigh), posterior thigh (PThigh), and pelvis.
_imu_sim.csv	200	Accel (m/s^2^), Gyro (degrees/s)	43	The data columns consist of pairs of six columns just as above (acceleration in the X, Y, Z direction and gyro in the X, Y, and Z direction). For this set there are seven virtual sensors, beginning with the pelvis and then alternating left and right for the thigh, shank, and foot.
_moment.csv	200	Newton-meters	11	The data columns are joint moments beginning with 3 for each hip (right then left; flexion/extension, adduction/abduction, rotation), 1 for each knee (right then left; flexion/extension), 1 for the ankle (right then left; dorsiflexion/plantarflexion).
_moment_filt.csv	200	Newton-meters	11	The data columns are in the same order as above as the moment with a lowpass zero-lag 5th order Butterworth filter at a cutoff frequency of 6 Hz.
_power.csv	200	Watts	11	The data columns are in the same order as above as the moment but for power.
_velocity.csv	200	degrees/s	13	The data columns are in the same order as above for the angle but for velocity.

### Raw data

The raw data are also contained in folders by participant. Within that folder there are several subfolders that contain different stages of processed data. The *CSV_Data* subfolder contains.csv files similar to the processed data but with all of the transitional data included. The *MarkerData* subfolder contains the marker data for each trial in a .trc format. The *ExtLoads* subfolder contains the ground reaction forces for each trial in a .mot format. The *Models* subfolder contains the different OpenSim models used for each trial and a Statics.csv table the specifies which model is associated with each task. These are based off different static trials in which specific marker locations may change if markers were replaced after falling off. The *Transforms* subfolder contains .mat files of tables for the transformation matrices from the global frame to the specific limb frame, which are used in simulating IMUs and pressure insoles.

### C3Ds

The c3d data is again separated in folders by participant. The *ViconData* subfolder within each participant contains the raw .c3d files for each task.

### Segmentation

The segmentation folder is also separated in folders by participant and contains the tasks that can be segmented (such as with the gait cycle or velocity thresholds) in their segmented form with the associated timestamps. Each participant folder contains two types of files, one with the task name and _parsing.mat that contains the timestamps used for segmentation of the data by right and left leg, and the other with the task name and _segmentation.mat that contains the angle, moment, filtered moment, velocity, and power segmented into cycles.

The rest of the folders in the dataset contain additional figures and videos as referenced above.

## Technical Validation

### Data collection

For data collection, the motion capture cameras were calibrated and verified to be within an acceptable error prior to data collection. In the case of a marker falling off during a trial, the marker was replaced and then a new static calibration was performed (the specific model that corresponds to each trial is given in RawData folder of the dataset). EMG’s were placed according to the Seniam guidelines^43^ and to verify placement, the signals were visually inspected while the participant performed movements to activate those muscles.

### Data processing

Inverse kinematics marker errors were visually examined to verify that markers were not mislabeled. Each task was examined to invalidate the inverse dynamics during segments where the participant’s external ground reaction forces were not captured. Inverse kinematics errors (as measured by the Euclidian distance from the motion capture location and the model location) above an 8 cm threshold were examined to determine the cause and corrected if possible. This is slightly higher than the error during normal gait analysis threshold^46^ due to the uniqueness of these activities. This was chosen based on visual inspection of true marker trajectories that resulted in IK errors maxing out near 8 cm. The average root mean squared marker error across tasks and participants for this dataset was 1.5 ± 0.2 cm. The orientation of the IMUs was verified by comparing the orientation of the gravity vector across participants during standing. Other signals were compared visually across participants to verify that a single participant was not an extreme outlier using plotting tools similar to those presented in Fig. 5.

### Comparison to public datasets

Many of the tasks included in this dataset are novel and unique tasks, however, some have been collected and published, specifically for the cyclic data. Comparing our dataset to that of Camargo *et al*. (speeds ranging from 0.5 m/s to 1.85 m/s) for level ground walking, the kinematics and kinetics are similar. For 1.8 m/s, both demonstrate sinusoidal hip kinematics and moments ranging from ~20° of hip flexion to 20° of extension with moment ranging from 1 Nm/kg of extension to 1 Nm/kg of flexion and hip power peaking around 2 W/kg. Knee kinematics range from 0° to 70° of flexion with a double peak and moments ranging from 0.5 Nm/kg of extension to 0.5 Nm/kg of flexion. Ankle kinematics range between 20° of dorsiflexion to 20° of plantarflexion and moment peaking around 1.5 Nm/kg of plantarflexion. Comparing to Reznick *et al*. (1.2 m/s), similar trends can be seen with the exception of smaller reported hip extension angles. This is similar for running with a slightly higher knee moment for level ground walking in our study as well as stairs and ramps.

Our dataset covers a range of walking speeds similar to previous datasets (0.6–1.8 compared to 0.5–1.85 m/s^15^ and ~0.28-1.1 m/s^25^ though with less granularity. Our running speeds were limited to a max speed of 2.5 m/s (compared to a max 4.5 m/s^11^) because our inclusion criteria did not involve being an active runner. One limitation of our work is that due to the rigorous nature of our protocol, this study recruited healthy young able-bodied adults (ranging from age 18–30 compared to previous larger ranges such as 19 to 67^9^).

### Supplementary information

Supplementary Table 1

## Data Availability

Code for simulating additional new IMUs for this dataset can be found in the code folder of the dataset and is based on the virtual IMUs included for each task^45^. A plotting script is included for visualizing the angle, moment, and power data from each of the tasks across participants. Finally, a function is included which groups the individual tasks by the groups used in the above analyses. Example scripts are included which demonstrate how to use each function.
